# Supplementary material for: Social predation in electric eels
Source: Ecol Evol. 2021 Jan 14;11(3):1088–92. doi: 10.1002/ece3.7121 (PMC7863634; doi:10.1002/ece3.7121)
Supplement: Supplementary file 9 — Supplementary Materials [file ECE3-11-1088-s009.docx]

**Supplementary materials for this manuscript include the following:**

Movies S1 to S8

**Movie S1**: First record of social predation in *Electrophorus voltai* during the low-water season in the mouth of a lake in the Iriri River drainage in 2012. 1). Group of electric eels (adult males and females; body length ranging from 1.2 to 1.8 meters) swimming in the hunting area; 2) A subset of electric eels (ca. 30 individuals) striking and disabling shoals of small fishes.

**Movie S2: Stage 1—Resting**: Electric eels surfacing to breathe (red arrows). 1) General view of resting area; 2) Close-up of electric eels gulping air, peacock bass cichlids (*Cichla melaniae*) can be seen swimming among eels. Camera 1 (Nikon D5100), real time (Figure 1B1).

**Movie S3: Stages 2 and 3—Interactions and Migration**: Electric eels swimming near the water surface. 1) Eels intraspecific interaction (duration 20 - 30 minutes); 2) Eels swimming together as a loose group migrating through a stretch of ~20 meters toward a shallow (<1 m deep) hunting area. 3) Eels migrating to the resting area after coordinated hunting. Camera 1 (Nikon D5100) accelerated x6, Camera 2 (Nikon D5100) accelerated x3 and Camera 3 (Nikon D5100) accelerated x4.5 (Figure 1B).

**Movie S4: Stage 4—Prey ball**: A prey ball is composed by shoals of small nektonic fishes, mostly characins (*Poptella* spp., *Moenkhausia* spp. and *Tetragonopterus* spp.). 1) Video frames indicating presence (red arrow)/absence of the prey ball (dark blotch) in the hunting area; 2) The shoals of fishes moving, inside the circle, during prey ball formation; 3) The same previous video with the prey ball highlighted (green blotch inside the circle). The effects were applied: black and white, high contrast, and Lumeri color (Changing the dark blotch to the green blotch), using Adobe Premiere Pro 2020.Camera 3 (GoPro 3+) accelerated x3 (Figure 1B4).

**Movie S5: Stage 4—Electric eels herding a prey ball**: Group of electric eels conducting the prey ball to the shallow area. Prey ball estimated from video clips to occupy an area ca. 12 m^2^. The coordinated driving movements are represented by three eels (tracked frame by frame using Adobe After Effects 2020). The green line represents the forward movement, circling or entering the center of the prey ball. The red line represents the reverse movement, usually leaving the center of the prey ball. Camera 3 (GoPro 3+) accelerated x7 (Figure 1B4).

**Movie S6: Stage 4—Electric eels attacking the prey ball**: The attacks usually start at the margin and extend until the middle of the channel. 1) High-voltage strikes showing preys jumping and falling stunned on the water surface; 2) High-voltage strikes inside the prey ball can be identified by the sinusoidal body posture (highlighted). Camera 3 (GoPro 3+) real time (Figure 1B4).

**Movie S7: Opportunistic predators (peacock bass cichlid**): Opportunistic behavior of peacock bass cichlids (*Cichla melaniae*) during coordinated hunting in electric eels. 1) Peeking at the prey ball; 2) Attacking stunned preys; 3) Peacock bass jumping when stroked by high-voltage discharges. Camera 3 (GoPro 3+) real time (Figure 1B4).

**Movie S8: Stage 4—Full coordinated hunting event:** Accelerate video from start to finish of coordinated hunting behavior (Stage 4): electric eels arriving at the hunting area (18h). Then, the eels begin herding and pushing the prey ball to the shallows. Different subgroups (ca. 5 to 50 individuals) make a series of five attacks between 18:17 and 18:35 h. After the attacks, the eels migrate back to the rest area at dusk (18:40 h). Group hunting occurred twice a day. Camera 3 (GoPro 3+) accelerated x41.8 (Figure 1B4).
